# Hemodynamic and Rhythmologic Effects of Push-Dose Landiolol in Critical Care—A Retrospective Cross-Sectional Study

**DOI:** 10.3390/ph16020134

**Published:** 2023-01-17

**Authors:** Sebastian Schnaubelt, Felix Eibensteiner, Julia Oppenauer, Daniel Tihanyi, Marco Neymayer, Roman Brock, Andrea Kornfehl, Christoph Veigl, Valentin Al Jalali, Sonja Anders, Barbara Steinlechner, Hans Domanovits, Patrick Sulzgruber

**Affiliations:** 1 Department of Emergency Medicine, Medical University of Vienna, 1090 Vienna, Austria; 2 Department of Pulmonology, Clinic Penzing, Vienna Healthcare Group, 1140 Vienna, Austria; 3 Department of Clinical Pharmacology, Medical University of Vienna, 1090 Vienna, Austria; 4 Department of Anaesthesia, Intensive Cate Medicine and Pain Medicine, Medical University of Vienna, 1090 Vienna, Austria; 5 Division of Cardiology, Department of Internal Medicine II, Medical University of Vienna, 1090 Vienna, Austria

**Keywords:** Landiolol, beta-blockers, critical care, intensive care, hemodynamic stability, dysrhythmia

## Abstract

Background: The highly β1-selective beta-blocker Landiolol is known to facilitate efficient and safe rate control in non-compensatory tachycardia or dysrhythmia when administered continuously. However, efficacy and safety data of the also-available bolus formulation in critically ill patients are scarce. Methods: We conducted a retrospective cross-sectional study on a real-life cohort of critical care patients, who had been treated with push-dose Landiolol due to sudden-onset non-compensatory supraventricular tachycardia. Continuous hemodynamic data had been acquired via invasive blood pressure monitoring. Results: Thirty patients and 49 bolus applications were analyzed. Successful heart rate control was accomplished in 20 (41%) cases, rhythm control was achieved in 13 (27%) episodes, and 16 (33%) applications showed no effect. Overall, the heart rate was significantly lower (145 (130–150) vs. 105 (100–125) bpm, *p* < 0.001) in a 90 min post-application observational period in all subgroups. The median changes in blood pressure after the bolus application did not reach clinical significance. Compared with the ventilation settings before the bolus application, the respiratory settings including the required FiO_2_ after the bolus application did not differ significantly. No serious adverse events were seen. Conclusions: Push-dose Landiolol was safe and effective in critically ill ICU patients. No clinically relevant impact on blood pressure was noted.

## 1. Introduction and Literature Review

### 1.1. A Favourable Pharmacological Profile

Landiolol is a highly cardioselective ultra short-acting (half-life (t_1/2_) 3–4.5 min) beta-blocker and has been primarily investigated for heart rate (HR) control in emergencies, intensive care, and perioperative settings [1,2,3,4]. It has been available in Japan for almost 20 years to treat non-compensatory supraventricular tachycardia and dysrhythmia [5,6]. In Europe, Landiolol has received approval for the same indications in 2016 [7,8]. In vivo testing revealed that it is nine times more potent in beta-blocking activity than the already well-known beta-blocker esmolol [4], and in vitro, an—at least—seven to eight times higher cardioselectivity [1,4] (ß1:ß2, 255:33 [4] or 216:30 [9]) has been observed than in esmolol. This cardioselectivity leads to a lower decrease in blood pressure (BP) than in other ß-blockers due to a lower selectivity towards ß2-adrenergic receptors, which are responsible for peripheral vasodilatation, and a more effective negative chronotropy through a higher selectivity towards cardiac ß1-receptors [4,10]. Overall, unselective ß-blockers are known for bronchoconstrictive effects by blocking ß2-receptors, with difficulties using them in patients with chronic pulmonary disease, or Raynaud’s phenomenon and peripheral vascular disease where blocking ß2-adrenergic receptors can worsen patients’ symptoms [11]. The reduction of BP in unselective ß-blockers is mainly driven by decreased cardiac output (reduction of HR and myocardial contractility). The newer generation of ß-blockers (third generation such as carvedilol, labetalol, and nebivolol) mediate vasodilatation via alpha-1 adrenoreceptor blockade and activation of nitric oxide (NO), and thus initiate a BP decrease [12]. Landiolol, however, shows no inhibitory effects towards L-type Ca^2+^-channels (and thus does not induce a change in myocardial action potential duration) or inward rectifier K^+^ channels (which are responsible for resting membrane potential maintenance). Moreover, Landiolol does not decrease plasma renin levels due to ß1-receptor blockage. All these implications contribute to the less negative hemodynamic or inotropic effect than, for instance, esmolol [13,14,15]. As an effect, the lethal dose of 50% (LD_50_) is 3.5 times higher than with esmolol [4].

Landiolol consists of an S-configurated hydroxyl and ester function, and therethrough the high cardioselectivity seems to be explainable. The short t_1/2_ can be attributed to the ethylene chain between the ester function and the phenyl ring [4]. Metabolism into an inactive metabolite 1 (M1) is initiated through hydrolysis of the ester chain, and further by serum pseudocholinesterase and carboxylesterase in the liver; the aforementioned short t_1/2_ is dosage independent [1,16]. Hereafter, M1 is metabolized into the more inactive M2-metabolite [17]. The affinity of the M1 metabolite for ß1-receptors is almost 40-fold lower than the active compound, which is of utmost importance for enabling a rapid clinical recovery after cessation of the administration if adverse effects should occur [1,9]. The t_1/2_ of the M1 metabolite (1.8 h) has been found to be similar between Caucasians and Asian ethnicities [2].

Landiolol exhibits no inverse agonistic effect but a very slight partial agonistic effect on ß1-receptors (biased agonism) via the elevation of cyclic adenosine monophosphate (cAMP). This partial agonistic effect is only observed in high concentrations of Landiolol, especially via phosphorylation of extracellular signal-regulated kinases 1/2 (ERK1/2) activated through the ß-arrestin signal pathway, which are involved in the downregulation of G-protein coupled receptors [9,18]. In chronic stimulation of the ß1-receptor activating the G-protein pathway, it is assumed that a cardiotoxic effect can be triggered and that the aforementioned described biased agonism stimulating the ß-arrestin pathway and suppressing G-protein signaling may be cardioprotective [19]. These effects have not been fully understood yet, in particular in relation to their clinical impact [9]. There is some evidence that extracellular-signal-regulated kinases (ERK) have protective effects on the progression of ischemia-induced reperfusion injury in the myocardium [20], which has also been seen in a rat animal model [21].

Furthermore, Landiolol does not show pharmacochaperoning, a phenomenon concerning continuous exposure to a ligand, resulting in increased receptor levels and an overshooting response to endogenous agonists once the treatment with the ligand is stopped. Without chaperoning, a rebound effect after infusion discontinuation is highly unlikely—as so observed in Landiolol [9]. An explanation for the lack of pharmacochaperoning in Landiolol can be a weaker permeability of the endoplasmic reticulum membrane due to the elevated size of the polar surface area when compared to esmolol: Landiolol is thus not able to reach sufficient intracellular concentrations and therefore sufficient levels of ß1-receptors on the surface of the cells cannot be achieved, and consequently, activation of ß1-receptors due to endogenous ligands is less common [9].

While Landiolol sufficiently induces HR reduction without the undesired impairment of the hemodynamic profile [22,23,24], it can also prevent a QT-interval prolongation [25] due to the potential impact of the ß1-agonism on the inhibition of delayed rectifier potassium currents (I_Kr_) which are responsible for ventricular repolarization. It is assumed that ß1-antagonism can initiate earlier repolarization [26,27]. Moreover, this has the potential of improving the imbalance of repolarization in patients with a risk of ventricular dysrhythmia, thus indicating a somewhat antiarrhythmic effect [25]: Several studies have reported a high potency of Landiolol of not only rate but also rhythm control in atrial fibrillation (AF) or atrial flutter (AFL), with surprisingly high conversion rates of up to 50–75% [28]. In detail, rhythm control occurred in almost 50% of post-cardiac-surgery and non-surgery (mostly myocardial infarction cases) ICU patients [29], and in nearly 70% in severe sepsis 24 h after application onset [30]. Some preliminary evidence suggests a higher efficacy in rhythmic tachycardia (atrial or ventricular) than in AF [1,30,31].

### 1.2. Effects on Morbidity and Mortality

Continuous Landiolol was shown to be safe in a variety of patient collectives, including AF and AFL with reduced left ventricular function [32,33], emergency department patients with AF [22], post-operative AF (POAF) [34], renal dysfunction [35,36,37], hepatic impairment [38], or sepsis-related tachyarrhythmia [39]. Landiolol can prevent POAF following cardiac thoracic surgery, with the additional effect of a reduced length of hospital stay [40]. A meta-analysis by Hao et al. confirmed the reduction of AF occurrence and found significantly lower overall adverse event rates, as well as lower mortality rates in patients treated with Landiolol after cardiothoracic surgery compared to standard of care [41].

Landiolol has been extensively researched in patients with impaired cardiac function (LVEF 25–50%); it was shown to perform better in rate control than digoxin [33]. Additionally, Landiolol seems to be applicable during percutaneous coronary intervention to prevent dysrhythmia and post-interventional myocardial injury [23], serving as a genuine alternative to class III antiarrhythmic drugs for the prevention of recurrent ventricular tachycardia or ventricular fibrillation [37,42]—again, also in patients with highly reduced cardiac function (LVEF < 25%) [43].

### 1.3. Landiolol and Inflammation

In a rat animal model, the immunomodulatory effects of Landiolol were observed via suppressing serum levels of high mobility group box-1 protein (HMGB-1), interleukin (IL-6), and tumor necrosis factor-alpha (TNF-alpha). These cytokines/proteins are relevant in inflammatory disease progression [44]. A current meta-analysis revealed a significantly lower 28-day mortality in patients with septic shock treated with cardioselective ß-blockers (esmolol or Landiolol), suggesting a potential class effect. In six of seven analyzed publications, esmolol was applied; only one study compared Landiolol with the standard of care [45]. There, a more favorable effect on HR reduction and inhibiting new-onset dysrhythmia was noted [24]. It is assumed that through the selective ß1-suppression, an exaggerated stimulation of ß2 adrenergic receptors occurs, which are expressed on the surface of CD4+ T-helper type 1 cells (Th1). The suppression of these Th1-cells leads to an increase in CD4+ T-helper 2 cells responsible for inhibiting macrophage activation, T-cell proliferation, and the release of pro-inflammatory cytokines. The activation of the ß2 pathway thus contributes to the anti-inflammatory effect at any phase of sepsis [46].

### 1.4. Bolus Application

The “push-dose” or “bolus” application of Landiolol has been first evaluated in perioperative settings. It has been reported as being effective in HR control with minor impairments on the hemodynamic profile [47,48]. The greatest effect of push-dose Landiolol on HR and BP (minor changes) was seen 5–7 min after injection with the most pronounced impact on HR and BP at the highest dosage regime (0.3 mg/kg). Therefore, a dose-dependency could be assumed (0.1–0.3 mg/kg) [47]. Another more recent investigation compared the Japanese formulation of Landiolol (Onoact^®^) to the European one (Rapibloc^®^) in healthy Caucasian volunteers, revealing a dose-dependent effect on HR after 1–3 min, and a minor dose-independent impact (<10 mmHg) on systolic BP within 3–12 min after injection [2]. When compared with a bolus application of esmolol, Landiolol leads to a faster and more prolonged HR control, and similar (<10 mmHg) but shorter BP impairment [17]. Using the bolus application, it can be assumed that the maximum HR reduction will be achieved faster than by a continuous infusion [14].

During computed tomography (CT) coronary angiography, patients with elevated HR and prior-taken oral ß-blockade, a bolus application of Landiolol for improving the CT image quality, showed effective HR control with no significant impact on systolic BP [49]. Moreover, it has been suggested that the rate-controlling response to a continuous Landiolol infusion in AF/AFL patients is inferior when a ß-blocker as a chronic co-medication is known [50]. These perceptions may be an important implication for the clinical use of Landiolol in patients with long-term ß-blockade.

Overall, little is known about the safety and efficacy of push-dose Landiolol in real-life critical care of patients at emergency departments (ED) or intensive care units (ICU). Especially regarding the hemodynamic and rhythmologic effects on critically ill patients with different tachycardia or dysrhythmia entities, an in-depth evaluation seems warranted. Therefore, we conducted a retrospective cross-sectional study at an ICU as a first step.

## 2. Materials and Methods

### 2.1. Study Population

**Figure 1:**
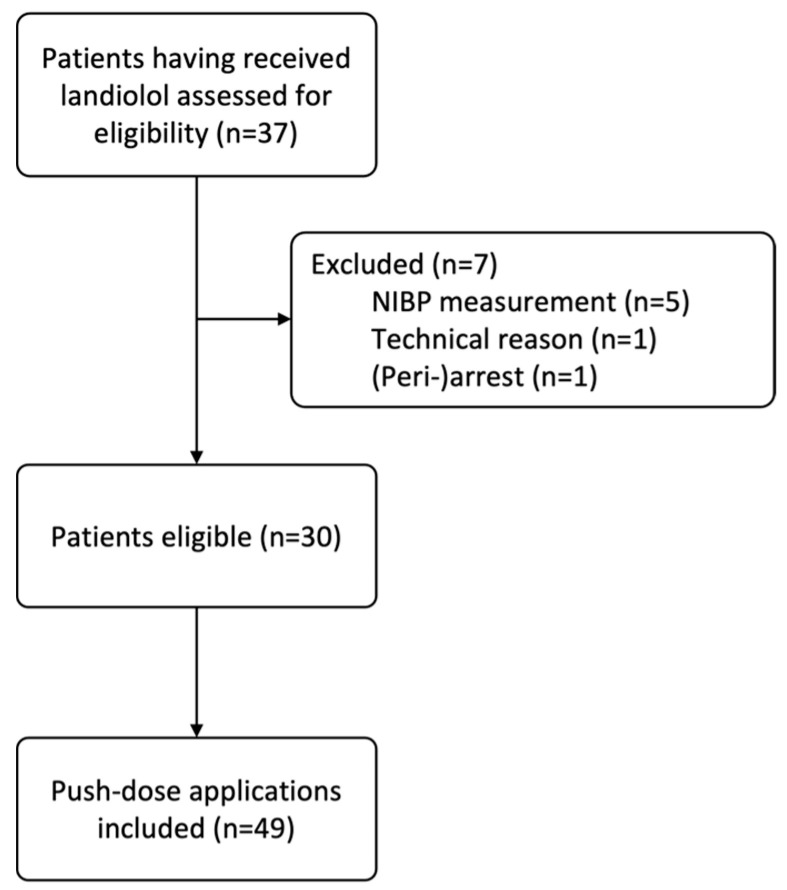
Flowchart of study inclusion. NIBP = non-invasive blood pressure.

In this retrospective cross-sectional study, we screened patients who were treated at the high-level ICUs of the Department of Pulmonology, Clinic Penzing, Vienna Healthcare Group, Austria, and the Department of Emergency Medicine, Medical University of Vienna, Austria, and who had received Landiolol (Rapibloc^®^) as a push-dose application at any time during their ICU stay between August 2020 and October 2021, independent of the reason of admission. Subsequently, all push-dose application episodes were evaluated. We enrolled patients above 18 years of age treated within the clear indication range of Landiolol (non-compensatory supraventricular tachycardia or non-compensatory sinus tachycardia) [7]. As exclusion criteria, we defined an age below 18 and missing invasive arterial pressure monitoring (IBPM). The study inclusion flowchart is seen in Figure 1.

### 2.2. Data Acquisition

All data were collected retrospectively through the respective electronic patient database system of the ICUs. Each eligible patient was checked for availability of full IBPM at 60 min before and 90 min after each push-dose application of Landiolol (some patients had received several applications with a maximum of four), and in this interval, BP values (every minute) and HR values (prior and post-push-dose) were collected by an information technology specialist.

### 2.3. Outcomes

The primary outcome was the BP (systolic, diastolic, mean arterial pressure (MAP)) before (60, 15, and 5 min), and after (5, 15, and 90 min) each push-dose application of Landiolol. A clinically relevant decrease in BP was defined as a decrease of over 10 mmHg after push-dose Landiolol. As secondary outcomes, we analyzed the efficacy of Landiolol towards rate or rhythm control, the potential impact on mechanical ventilation parameters, and the potential influence of catecholamine use, as well as the potential impact of previously administered oral beta-blockers. We also searched for adverse events related to Landiolol regarding clinically relevant hypotension, dysrhythmia, or allergic reactions. Successful rate control was defined as a reduction of the HR above 15%; successful rhythm control was determined as a conversion to a sinus rhythm at least for 20 min.

We also analyzed subgroups divided in gender, regular tachycardia (RT), or irregular tachycardia (IRT) before the bolus application and the fluid balance of the patient above or below 900 mL. Regarding the fluid balance, at the ICU where the bolus was administered, the fluid balance is reset to zero every 24 h at 6 o´clock in the morning.

### 2.4. Statistical Analysis

Numeric data are expressed as medians with corresponding interquartile ranges (IQR). They were compared using the Kruskal–Wallis test or Wilcoxon rank-sum test. Categorical data are presented as counts and percentages and were compared via chi-square or Fisher exact test as appropriate. Linear regression was performed on all parameters and for the significant parameters, a multiple linear regression model with stepwise backward model selection based on the Bayesian information criteria (BIC) was applied to test the impact of these parameters on the MAP 15 min after bolus injection. All tests were two-sided, and *p*-values < 0.05 were considered statistically significant. Statistical analyses were performed using SPSS 27.0 (IBM, Armonk, NY, USA).

## 3. Results

### 3.1. Patients and Boli

Thirty patients with sudden onset of non-compensatory supraventricular tachycardia were investigated in this trial (Figure 1). The maximum number of applications was four individual boli of Landiolol in one patient. The median patient age was 67 (55–72) years, and 57% were men. A third (33%) of the patients had been admitted to the ICU due to respiratory failure, 37% for weaning following a COVID-19 infection, and 10% were post-cardiac-arrest patients. The studied 30 patients had received a total of 49 bolus applications of Landiolol, with a median dosage of 7 (6–13) mg. Overall, 27 cases of tachycardia were with IRT (atrial fibrillation), and 22 were RT (atrial flutter or other). At the time point of tachycardia onset, patients with IRT were being treated with catecholamines significantly more often (*p* = 0.008), and they had a more positive fluid balance (*p* = 0.089) than the patients with RT. Overall, further details on comorbidities, admission, ventilation status, and medication are mentioned in Table 1 and Table 2. Data on diagnostic imaging and laboratory results are presented descriptively in Supplementary Table S8.

### 3.2. Outcomes

**Figure 2:**
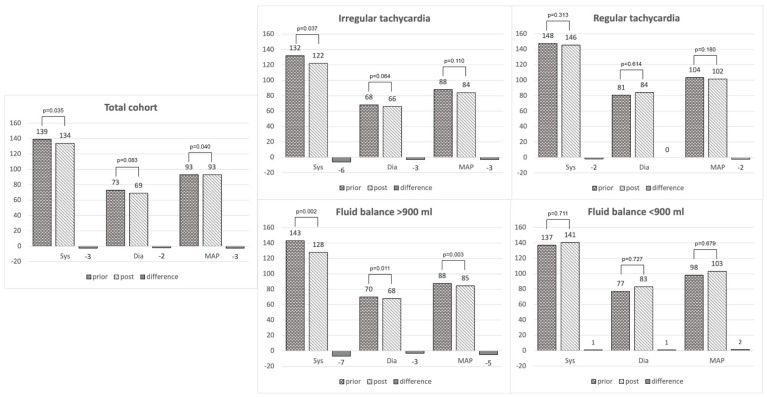
Hemodynamic profiles pre- and post-five minutes after push-dose Landiolol application. Blood pressure values are stated as medians (sys = systolic, dia = diastolic, MAP = mean arterial pressure) and are given in millimeters mercury (mmHg). The fluid balance accounts for the overall balance right before push-dose Landiolol application. “Irregular tachycardia” and “regular tachycardia” concerns the heart rhythm before the bolus application.

**Figure 3:**
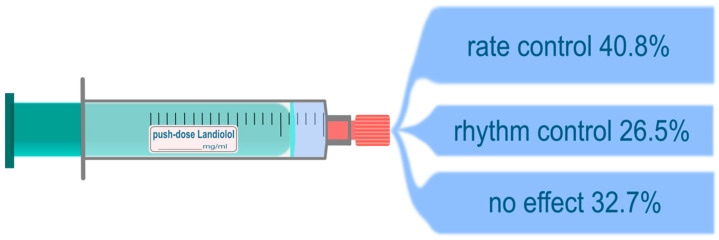
The performance of push-dose Landiolol in rate and rhythm control in the overall study cohort.

**Figure 4:**
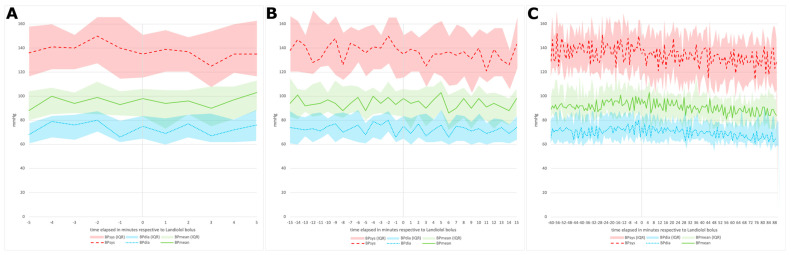
Hemodynamic profiles (**A**) five minutes pre- and post-Landiolol bolus application, (**B**) 15 min pre- and post-, and (**C**) 60 min pre- and 90 min post-, respectively.

Figure 2, Figure 3 and Figure 4 depict the measured outcomes.

#### 3.2.1. Heart Rate

Successful HR control was accomplished in a total of 20 (41%) cases, rhythm control was achieved in 13 (27%) episodes, and 16 (33%) applications showed no effect (Figure 3). Overall, the HR was significantly lower after the application of Landiolol (145 (130–150) vs. 105 (100–125) bpm, *p* < 0.001) in a 90 min post-application observational period; this was also observed throughout all subgroups (Supplementary Table S1). The frequency of regular tachycardia was significantly lower than in patients with irregular tachycardia (130/min vs. 150/min, *p* = 0.004) (Table 2). A chronic oral beta-blocker intake did not influence the effect of Landiolol on the HR before the bolus (*p* = 0.424), and after the bolus (*p* = 0.437), or on the combined endpoint of successful rhythm or rate control and the response (*p* = 0.651).

#### 3.2.2. Blood Pressure

Overall, the systolic BPs and MAPs 5 and 15 min prior to the bolus application of Landiolol were significantly lower than 5 and 15 min afterward, whereas the diastolic BP did not significantly differ. When further evaluating this, we found that in cases with a fluid balance >900 mL, the BP significantly differed, whereas in a fluid balance of <900 mL, this was not the case. In patients being IRT at the bolus timepoint, only the systolic BP differed significantly afterward when compared to before. Of importance, the median differences in all mentioned situations amounted to vanishingly small numbers and did not reach clinical significance (Figure 2).

The detailed BP results before and after the bolus application in all cases and throughout the subgroups are shown in Supplementary Tables S4–S8. In Figure 4, hemodynamic profiles of pre- and post-Landiolol bolus are shown graphically over various time periods (5 min, 15 min, and 60/90 min, respectively).

In the multiple linear regression, we adjusted for age, prior/post fraction of inspired oxygen (FiO_2_), prior total inspiratory pressure (Pinsp), prior pressure mean (Pmean), catecholamines, HR control, RT, a prior enlarged cardiac silhouette, and the median MAP 15 min before the bolus application. These covariates were evaluated in a simple linear regression (Supplementary Table S2). However, only the median MAP 15 min prior to the bolus application was a significant impact factor on the BP after the bolus application (*p* < 0.001) (Table 3). In the linear regression model, a previous catecholamine treatment was a significant impact factor on the MAP after the bolus application (*p* = 0.017); however, a previous chronic oral beta-blocker intake had no significant impact on BP changes (*p* = 0.481).

#### 3.2.3. Respiration

Compared with the ventilation settings before the bolus application, the respiratory settings including the required FiO_2_ after the bolus application did not differ significantly, and no adverse respiratory events were observed (Supplementary Table S3).

#### 3.2.4. Adverse Events

No adverse events such as persistent hemodynamic instability, allergic reaction, dysrhythmia, respiratory failure, or death were observed. Only in two cases, a BP decline of more than 20% was observed. These declines did not need any circulatory support and recovered without any intervention. It could not be clarified if the bolus application was causal.

## 4. Discussion

In this retrospective, cross-sectional study, we observed no clinically relevant decrease in BP (<5%) due to a bolus application of Landiolol in critical care patients with supraventricular tachycardia. Furthermore, Landiolol showed not only an effect on HR control but also a surprisingly strong effect on rhythm control as well. The findings in our analysis correspond with previous studies of continuous Landiolol application with limited effect on BP [22,23,24]. A small amount of literature about Landiolol’s more recent push-dose formulation is available. Only a few studies investigated the effect of push-dose Landiolol on hemodynamics and HR or rhythm control. Of note, no study is available on critically ill ICU patients.

Harasawa et al. studied 32 patients who received between 0.1–0.3 mg/kg body weight (BW) of Landiolol, and they observed a decrease in BP of about 15% at the highest dosage in patients under general anesthesia with intracranial and maxillofacial tumors [47]. In a small study of healthy participants, the BP decreased by a maximum of 8.5%, even at the same dosage levels of 0.1–0.3 mg/kg BW [17]. In a radiology study using Landiolol (0.125 mg/kg BW) as a pre-treatment for HR reduction for better image quality, the BP stayed almost stable (<2% change in systolic BP). Nevertheless, the BP remained stable although they received other BP medication (metoprolol, propranolol, verapamil) before the Landiolol injection [49]. In this case, a stronger effect on the decrease of BP might have been expected. Compared to our study, in which we evaluated ICU cases where 93% of patients were under mechanical ventilation support and 39% received catecholamines, the patients in the above-mentioned studies had probably been healthier and were potentially at lower risk for hemodynamic and respiratory deterioration.

Interestingly, patients with a higher fluid balance seemed to be at greater risk for declining BP after applying Landiolol. A potential explanation could be a more inferior hemodynamic and more critically ill state of the patients. Therefore, they were treated with higher amounts of fluid for hemodynamic stabilization. Another explanation for this effect could be the more inferior kidney function in critically ill patients, leading to acute kidney injury with subsequent fluid overload [51,52]. It is known that a higher fluid balance is associated with a more severe medical condition and an increased risk of mortality [53,54,55].

Regarding the effects on HR and rhythm, our study observed a clinically significant HR control (145/min to 105/min, *p* < 0.001). To our knowledge, our study is the first that investigated the rhythm control effect of a Landiolol bolus application. We could observe a “Bolus Rule of Thirds” in our study collective: In about one-third of cases for each, a sustained HR control, a successful conversion into a sinus rhythm, and no effect was seen. For the continuous formulation of Landiolol, a high rate of rhythm control of almost 50% in post-surgical and internal ICU patients has been described before [29]. Okajima et al. investigated 39 septic patients with supraventricular tachycardia and infused continuous Landiolol. Within one hour after the start of infusion, 25% of patients achieved a sinus rhythm, and within 24 h almost 70% converted to a sinus rhythm [30]. This cohort is the most comparable to ours, and they achieved almost the same success of rhythm control with continuous infusion as we could observe in push-dose Landiolol. The overall successful effect (rhythm or HR control combined) was 67%, and 33% showed no effect after the Landiolol bolus. Interestingly, we found no significant influence of previous chronic beta-blocker medication on whether the bolus led to an effect or not. In further studies it could be interesting why almost one-third of the arrhythmias were not affected by Landiolol injection, and to evaluate the reason for this finding.

Acute respiratory failure is one of the most common reasons for admission to an ICU [56,57]. Even in our mixed ICU collective, it was the second most common reason for ICU admission. Especially in respiratorily compromised patients, the application of ß-blockers is still discussed controversially due to concerns about the deterioration of respiratory failure or pre-existing pulmonary disease [58]. Kargin et al. investigated older generations of ß-blockers (bisoprolol, metoprolol, and carvedilol) in patients with chronic obstructive pulmonary disease (COPD) and respiratory failure for HR control. They evaluated in-hospital mortality and 30-day mortality after discharge and found no difference compared with a control group that used diltiazem, digoxin, or amiodarone for HR control [59]. In a meta-analysis, the effect of ß-blockers on the forced expiratory volume in 1 s (FEV1) was not reduced and no higher numbers of exacerbation of COPD were observed [60]. The newer generations of intravenous cardioselective ß1-blockers as Landiolol and esmolol seemed to have a protective pulmonary effect due to a reduction of blood flow in the vascular system of the lungs and a decrease of endothelial pulmonary cell damage [44,61]. These findings combined with our analysis can serve as a reference for future studies investigating the effect of highly cardioselective ß1-blockers on pulmonary function. Of importance, however, is that our findings emphasize the respiratory safety profile of push-dose Landiolol.

Another rather important aspect is the costs of intravenously administered therapies, which are often quite expensive. Walter et al. compared the cost-effectiveness between Landiolol and standard of care (e.g., other ß-blockers or diltiazem) in preventing the onset of post-operative atrial fibrillation (POAF). The results elucidated a cost reduction in applying Landiolol compared to the standard of care [62]. This economic sight of health care is often neglected, but it should be a considerable part of the decision process if comparable treatments are applied.

In terms of clinical applicability, supraventricular tachycardia—including arrhythmias such as sinus tachycardia, atrial fibrillation, atrial tachycardia, atrioventricular nodal re-entrant tachycardia (AVNRT), atrioventricular re-entrant tachycardia (AVRT), etc. [63]—regularly occurs in emergency or intensive care medicine. Short-acting ß-blockers such as esmolol or metoprolol are already recommended as a treatment option for HR control in RT without signs of decompensated heart failure [63]. Also in patients with IRT, ß-blockers are the first line of therapy for HR control in preserved or reduced LVEF, as well as in critically ill patients with a low probability of successful rhythm control [31]. In our study, however, we only included supraventricular tachycardia, mainly consisting of atrial fibrillation, non-compensatory sinus tachycardia, or atrial flutter.

Our trial has several limitations. First, it was a retrospective study. For future studies, a randomized controlled trial should be planned and at least two standardized treatment arms for better comparison are necessary. Second, a minor limitation especially regarding BP changes was that the dosage of the catecholamines was not available in detail because the used perfusors did not automatically transfer data into the intensive care information system. Due to the necessity of manual documentation, only some changes in the dosage of catecholamines were noted exactly. Minor changes were retrospectively adapted every one hour; this could have led to an inaccurate appraisal of catecholamine effects. Third, we did not follow up with the patients for in-hospital or out-of-hospital mortality data and long-term outcomes. Fourth, we could not evaluate exactly when the last (chronic) oral beta-blockers prior to the Landiolol bolus were taken or given; therefore, the previously suggested inferior effect on rate control in patients on chronic oral beta-blockers cannot be reported in our study [50].

## 5. Conclusions

To the best of our knowledge, this was the first study to evaluate the hemodynamic, rhythmologic, and respiratory effects of push-dose Landiolol in critically ill patients. Push-dose Landiolol seemed safe without a clinically relevant impact on BP, and no adverse events occurred. Furthermore, push-dose Landiolol showed good efficacy in rate and rhythm control. It is still unclear which factors influence the successful response to Landiolol. An algorithm for the bolus application of Landiolol in critically ill and emergency department patients could be implemented in the in- and pre-hospital setting after further investigation.

## Figures and Tables

**Table 1:** Patients’ basic demographics, overall and stratified in terms of gender. The fluid balance accounts for the overall balance right before push-dose Landiolol application. “IRT” and “RT” concerns the heart rhythm before bolus application. Categorical data are presented as counts and percentages, and continuous data as medians and interquartile ranges (IQRs). Categorical data were analyzed using a test for linear association (Mantel–Haenszel chi-square test) or Fisher’s exact test, and continuous data using Kruskal–Wallis test for testing within the subgroups. Wilcoxon rank test was used for analyzing blood pressure and heart rate changes. RT = regular tachycardia, IRT = irregular tachycardia, BMI = body mass index; AHTN = arterial hypertension; HLP = hyperlipidemia; DM = diabetes mellitus; CAD = coronary artery disease; CKI = chronic kidney injury; PAD = peripheral arterial disease; AF = atrial fibrillation; AFL = atrial flutter; CPR = cardiopulmonary resuscitation; COVID = coronavirus disease; ICU = intensive care unit.

(n = Patients)	Total	Male	Female	*p*-Value	RT	IRT	*p*-Value	Fluid Balance < 900 mL	Fluid Balance > 900 mL	*p*-Value
**N** (% of total)	30	17 (56.7)	13 (43.3)		16 (53.3)	14 (46.7)		14 (46.7)	15 (50.0)	
Male sex, n (%)	17 (56.7)				11 (68.8)	6 (42.9)	0.153	9 (64.3)	7 (46.7)	0.340
Age, years (IQR)	67 (55–72)	67 (55–71)	67 (55–77)	0.630	66 (53–73)	70 (58–72)	0.492	59 (44–71)	69 (62–77)	0.066
BMI, kg/m^2^ (IQR)	29 (23–31)	24.2 (22.7–30.1)	30.5 (29.4–36.2)	**0.038**	28.9 (23.1–35.3)	29.3 (22.5–30.6)	0.406	28.3 (23.2–37.7)	29.4 (22.7–30.9)	0.458
Height, cm (IQR)	171 (165–180)	180 (171–182)	165 (160–170)	**<0.001**	177 (169–182)	169 (162–180)	0.086	178 (165–182)	170 (164–180)	0.282
Weight, kg (IQR)	80 (70–96)	80 (70.0–97.5)	85 (71.5–95.0)	0.629	87.5 (71.3–107.5)	78.5 (64.8–90.0)	0.095	85.0 (70.0–111.8)	80 (75–90)	0.405
**Comorbidities**										
AHTN, n (%)	12 (40)	5 (29.4)	7 (53.8)	0.176	7 (43.8)	5 (35.7)	0.654	6 (42.9)	6 (40.0)	0.876
HLP, n (%)	3 (10)	2 (11.8)	1 (7.7)	0.713	3 (18.8)	0 (0)	0.228	2 (14.3)	1 (6.7)	0.598
DM II, n (%)	10 (33.3)	4 (23.5)	6 (46.2)	0.255	6 (37.5)	4 (28.6)	0.709	5 (35.7)	5 (33.3)	1.000
CAD, n (%)	6 (20.0)	3 (17.6)	3 (23.1)	1.000	3 (18.8	3 (21.4)	1.000	2 (14.3)	4 (26.7)	0.651
CKI, n (%)	4 (13.3)	1 (5.9)	3 (23.1)	0.290	1 (6.3)	3 (21.4)	0.315	3 (21.4)	1 (6.7)	0.330
PAD, n (%)	2 (6.7)	0 (0)	2 (15.4)	0.179	1 (6.3)	1 (7.1)	1.000	1 (7.1)	1 (6.7)	1.000
Thyroid disease, n (%)	8 (26.7)	3 (17.6)	5 (38.5)	0.242	4 (25.0)	4 (28.6)	1.000	4 (28.6)	4 (26.7)	1.000
AF, n (%)	13 (43.3)	5 (29.4)	8 (61.5)	0.078	3 (18.8)	10 (71.4)	**0.004**	5 (35.7)	8 (53.3)	0.340
Paroxysmal, n (%)	12 (40.0)	5 (29.4)	7 (53.8)	0.176	3 (18.8)	9 (64.3)	**0.011**	5 (35.7)	7 (46.7)	0.550
Permanent, n (%)	1 (3.3)	0 (0)	1 (7.7)	0.433	0 (0)	1 (7.1)	0.467	0 (0)	1 (6.7)	1.000
AFL, n (%)	2 (6.7)	1 (7.7)	1 (7.7)	1.000	1 (6.3)	1 (7.1)	1.000	2 (14.3)	0 (0)	0.224
**Reason for admission**										
Neurological reason, n (%)	1 (3.3)	1 (5.9)	0 (0)	1.000	0 (0)	1 (7.1)	0.467	0 (0)	1 (6.7)	1.000
Dysrhythmia, n (%)	1 (3.3)	1 (5.9)	0 (0)	1.000	0 (0)	1 (7.1)	0.467	1 (7.1)	0 (0)	0.483
Respiratory failure, n (%)	10 (33.3)	6 (35.3)	4 (30.8)	1.000	5 (31.3)	5 (35.7)	1.000	5 (35.7)	4 (26.7)	0.700
St.p. CPR, n (%)	3 (10)	1 (5.9)	2 (15.4)	0.565	1 (6.3)	2 (14.3)	0.586	0 (0)	3 (20.0)	0.224
Weaning post-COVID, n (%)	11 (36.7)	6 (35.3)	5 (38.5)	1.000	8 (50)	3 (21.4)	0.105	7 (50)	4 (26.7)	0.196
Sepsis, n (%)	2 (6.7)	1 (5.9)	1 (7.7)	1.000	1 (6.3)	1 (7.1)	1.000	0 (0)	2 (13.3)	0.483
Other reasons, n (%)	2 (6.7)	1 (5.9)	1 (7.7)	1.000	1 (6.3)	1 (7.1)	1.000	1 (7.1)	1 (6.7)	1.000
**Admission from...**										
home, n (%)	7 (23.3)	3 (17.6)	4 (30.8)	0.666	3 (18.8)	4 (28.6)	0.675	2 (14.3)	4 (26.7)	0.651
General ward, n (%)	8 (26.7)	4 (23.5)	4 (30.8)	0.698	2 (12.5)	6 (42.9)	0.101	1 (7.1)	7 (46.7)	**0.035**
ICU, n (%)	15 (50)	10 (58.8)	5 (38.5)	0.269	11 (68.8)	4 (28.6)	**0.028**	11 (78.6)	4 (26.7)	**0.005**

**Table 2:** Details and circumstances of Landiolol bolus application, overall and stratified in terms of gender, RT and IRT, and fluid balance. The fluid balance accounts for the overall balance right before push-dose Landiolol application. “IRT” and “RT” concerns the heart rhythm before bolus Landiolol application. Categorical data are presented as counts and percentages, and continuous data as medians and interquartile ranges (IQRs). Categorical data were analyzed using a test for linear association (Mantel–Haenszel chi-square test) or Fisher’s exact test, and continuous data using Kruskal–Wallis test for testing within the subgroups. Wilcoxon rank test was used for analyzing blood pressure and heart rate changes. RT = regular tachycardia; IRT = irregular tachycardia; NIV = non-invasive ventilation; NHF = nasal high-flow.

(n = Bolus Applications)	Total	Male	Female	*p*-Value	RT	IRT	*p*-Value	Fluid Balance < 900 mL	Fluid Balance > 900 mL	*p*-Value
**N** (% of total)	49	26 (53.1)	23 (46.9)		22 (44.9)	27 (55.1)		19 (38.8)	28 (57.1)	
Length of stay until Landiolol bolus, days (IQR)	13 (6–22)	14 (6–25)	11 (5–22)	0.569	13 (8–25)	14 (4–22)	0.656	14 (7–20)	16 (4–26)	0.712
Dose of Landiolol, mg (IQR)	7.0 (6.5–13.0)	7 (6–13)	7 (7–13)	0.430	7 (6.8–10.8)	7 (6–14)	0.551	7 (7–14)	7 (7–11.5)	0.404
Irregular tachycardia, n (%)	27 (55.1)	11 (42.3)	16 (69.6)	0.056				7 (36.8)	19 (67.9)	**0.038**
HR prior bolus, bpm (IQR)	145 (130–150)	145 (129–153)	150 (130–150)	0.569	130 (125–150)	150 (140–155)	**0.004**	150 (135–160)	143 (126–150)	0.080
HR post bolus, bpm (IQR)	105 (100–125)	113 (100–125)	100 (95–130)	0.385	105 (99–121)	105 (100–130)	0.859	115 (95–130)	105 (100–125)	0.472
Successful Landiolol response, n (%)	33 (67.3)	18 (69,2)	15 (65.2)	0.765	16 (27.7)	17 (63.0)	0.468	12 (63.2)	19 (67.9)	0.739
Rate control, n (%)	20 (40.8)	11 (42.3)	9 (39.1)	0.821	12 (54.5)	8 (29.6)	0.078	7 (36.8)	13 (46.4)	0.541
Rhythm control, n (%)	13 (26.5)	7 (26.9)	6 (26.1)	0.947	4 (18.2)	9 (33.3)	0.232	5 (26.3)	6 (21.4)	0.737
No effect, n (%)	16 (32.7)	8 (30.8)	8 (34.8)	0.765	6 (27.3)	10 (37.0)	0.468	7 (36.8)	9 (32.1)	0.739
Switch to perfusor, n (%)	11 (22.4)	8 (30.8)	3 (13.0)	0.138	2 (9.1)	9 (33.3)	**0.043**	3 (15.8)	7 (25.0)	0.718
Offset										
Electrical cardioversion, n (%)	2 (4.1)	1 (3.8)	1 (4.3)	0.929	0 (0)	2 (7.4)	0.495	2 (10.5)	0 (0)	0.158
Pharmacological cardioversion, n (%)	28 (57.1)	14 (53.8)	14 (60.9)	0.620	12 (54.5)	16 (59.3)	0.779	12 (63.2)	14 (50)	0.373
Spontaneous, n (%)	19 (38.8)	10 (38.5)	9 (39.1)	0.962	9 (40.9)	10 (37.0)	0.783	5 (26.3)	14 (50)	0.104
Fluid balance, ml (IQR)	1340 (60–1979)	1210 (138–1727)	1540 (−240–2590)	0.371	480 (−150–1835)	1520 (630–2297)	0.089	n.a.	n.a.	n.a.
**Ventilation**										
Mechanical ventilation, n (%)	46 (93.9)	23 (88.5)	23 (100)	0.237	20 (90.9)	26 (96.3)	0.581	17 (89.5)	27 (96.4)	0.338
NIV, n (%)	5 (10.2)	3 (11.5)	2 (8.7)	1.000	2 (13.6)	2 (7.4)	0.474	1 (5.3)	3 (10.7)	0.638
Invasive, n (%)	31 (63.3)	20 (76.9)	11 (47.8)	**0.035**	14 (63.6)	17 (63.0)	0.961	11 (57.9)	19 (67.9)	0.485
NHF, n (%)	10 (20.4)	0 (0)	10 (43.5)	**<0.001**	3 (13.6)	7 (25.9)	0.242	5 (26.3)	5 (17.9)	0.366
**Medication**										
Catecholamine support, n (%)	19 (38.8)	9 (34.6)	10 (43.5)	0.569	4 (18.2)	15 (55.6)	**0.008**	4 (21.1)	14 (50)	**0.045**
Corticosteroids, n (%)	21 (42.9)	11 (42.3)	10 (43.5)	0.934	11 (50)	10 (37.0)	0.362	8 (42.1)	11 (39.3)	0.847
Antibiotics, n (%)	40 (81.6)	22 (84.6)	18 (78.3)	0.716	17 (77.3)	23 (85.2)	0.713	15 (78.9)	24 (85.7)	0.697
Chronic oral ß-blocker, n (%)	13 (26.5)	8 (30.8)	5 (21.7)	0.607	7 (31.8)	6 (22.2)	0.672	5 (26.3)	7 (25.0)	0.987

**Table 3:** Multiple linear regression for the MAP 15 min after the bolus application of Landiolol. Adjusted for covariates in a backward multiple regression for age, FiO_2_ prior, Pinsp prior, Pmean prior, FiO_2_ post, Pinsp post, Pmean post, catecholamine, rate control, regular tachycardia, enlargement of the cardiac silhouette prior, mean MAP 15 min prior.

	Blood Pressure MAP 15 min Post			
Predictors	Coefficient	SE	CI	*p*-Value
Constant	14.008	13.750	−14.114–42.130	0.317
FiO_2_ prior	−0.188	0.096	−0.384–0.007	0.058
Mean MAP 15 min prior	0.912	0.108	0.690–1.134	**<0.001**

## Data Availability

All data are available upon reasonable request to the corresponding author.

## Supplementary Materials

The following supporting information can be downloaded at: https://www.mdpi.com/article/10.3390/ph16020134/s1. Supplemental Tables S1–S8. Supplementary Table S1: Differences in heart rate prior- and post Landiolol bolus application in various subgroups. Supplementary Table S2: Simple linear regression analyzing covariates on MAP 15 min after bolus application. Supplementary Table S3: Differences in respiratory parameters prior- and post Landiolol bolus application in various subgroups. Supplementary Table S4: Differences in blood pressure prior- and post Landiolol bolus application (subdivided in 5 min, 15 min and 60 to 90 min before and after application). Supplementary Table S5: Differences in blood pressure prior- and post Landiolol bolus application (subdivided in 5 min, 15 min and 60 to 90 min before and after application) in the subgroup gender. Supplementary Table S6: Differences in blood pressure prior- and post Landiolol bolus application (subdivided in 5 min, 15 min and 60 to 90 min before and after application) in the subgroup of regular and irregular tachycardia before the bolus. Supplementary Table S7: Differences in blood pressure prior- and post Landiolol bolus application (subdivided in 5 min, 15 min and 60 to 90 min before and after application) in the subgroups fluid balance above 900 mL und below 900 mL. Supplementary Table S8: Differences in diagnostic imaging (X-ray) prior- and post-Landiolol bolus and latest cardiac function (LVEF) prior Landiolol bolus in various subgroups.
