# Supplementary material for: Hemodynamic and Rhythmologic Effects of Push-Dose Landiolol in Critical Care—A Retrospective Cross-Sectional Study
Source: Pharmaceuticals (Basel). 2023 Jan 17;16(2):134. doi: 10.3390/ph16020134 (PMC9967759; doi:10.3390/ph16020134)
Supplement: Supplementary file 1 [file pharmaceuticals-16-00134-s001.zip › pharmaceuticals-2150789-supplementary.pdf]

Article

Supplementary Materials

Supplementary Table S1. Differences in heart rate prior- and post Landiolol bolus application in various subgroups. The fluid balance accounts for the overall balance right before push-dose Landiolol application. "RT" and "IRT" concerns the heart rhythm before bolus application. Data are presented as medians and interquartile ranges (IQRs) and analyzed using Wilcoxon-rank test. RT = regular tachycardia, IRT = irregular tachycardia.

Suppl. Table S1	Heart rate prior bolus, bpm (IQR)	Heart rate post bolus, bpm (IQR)	p-value
Total	145 (130-150)	105 (100-125)	**< 0.001**
Male	145 (129-153)	113 (100-125)	**< 0.001**
Female	150 (130-150)	100 (95-130)	**< 0.001**
RT	130 (125-150)	105 (99-121)	**< 0.001**
IRT	150 (140-155)	105 (100-130)	**< 0.001**
Fluid balance > 900ml	143 (126-150)	105 (100-125)	**< 0.001**
Fluid balance < 900ml	150 (125-160)	115 (95-130)	**0.001**
No effect	143 (120-150)	140 (125-150)	0.501
Rhythm control	150 (130-168)	95 (80-100)	**0.001**
Rate control	145 (130-150)	105 (100-114)	**< 0.001**

Supplementary Table S2 Simple linear regression analyzing covariates on MAP 15 minutes after bolus application. BMI = Body mass index, NIV = non-invasive ventilation, NHF = nasal high flow, FiO2 = fraction of inspired oxygen, Pinsp = total inspiratory pressure, Pmean = pressure mean, PEEP = post end-expiratory pressure, RR = respiratory rate, HR = heart rate, LVEF = left ventricular ejection fraction, MAP = mean arterial pressure.

Suppl. Table S2	Median MAP 15 min post Bolus			
**Predictors**	**Coefficient**	**SE**	**CI**	**p-value**
Gender	-5.768	5.152	-16.132 – 4.597	0.269
Age	-0.434	0.150	-0.735 – - 0.133	**0.006**
BMI	0.501	0.399	-0.301 – 1.303	0.215
**Ventilation**				
Mechanical ventilation at bolus application	-17.496	10.563	-38.746 – 3.753	0.104
NIV	1.884	8.602	-15.421 – 19.189	0.828
Intubation	-4.872	5.253	-15.439 – 5.696	0.358
Trachestomy	3.870	6.218	-8.640 – 16.379	0.537
Invasive	-2.165	5.395	-13.018 – 8.688	0.690
NHF	-4.156	6.436	-17.103 – 8.790	0.522
FiO2 prior	-0.637	0.119	-0.879 – -0.396	**< 0.001**
RR prior	-0.008	0.345	-0.716 – 0.701	0.983
Pinsp prior	-1.395	0.448	-2.306 – -0.485	**0.004**
Pmean prior	-2.970	0.985	-4.972 – -0.968	**0.005**
PEEP prior	-0.870	1.621	-4.164 – 2.423	0.595
FiO2 post	-0.579	0.112	-0.804 – -0.353	**< 0.001**
RR post	0.204	0.527	-0.892 – 1.300	0.703
Pinsp post	-1.638	0.510	-2.679 – -0.598	**0.003**
Pmean post	-3.062	0.881	-4.861 – -1.263	**0.002**
PEEP post	-1.418	1.605	-4.687 – 1.851	0.384
**Medication**				

Catecholamines	-12.444	5.029	-22.562 – -2.326	**0.017**
Corticosteroids	-1.048	5.262	-11.634 – 9.539	0.843
Antibiotics	-4.808	6.691	-18.270 – 8.653	0.476
Oral ß-blockers	4.391	6.171	-8.054 – 16.835	0.481
Dosage of oral ß-blockers	1.422	1.584	-1.787 – 4.631	0.375
**Bolus details**				
Periode of hospitalization until bolus	0.117	0.166	-0.216 – 0.451	0.482
Dosage landiolol	-0.288	0.592	-1.364 – 0.788	0.592
HR prior	-0.112	0.145	-0.403 – 0.179	0.442
HR post	0.044	0.115	-0.187 – 0.276	0.702
Rate control	12.785	4.962	2.804 – 22.767	**0.013**
Rhythm control	-13.277	5.574	-24.490 – -2.063	0.021
No effect	-2.277	5.546	-13.433 – 8.880	0.683
Any effect	2.277	5.546	-8.880 – 13.433	0.683
Switch to perfusor	-7.267	6.153	-19.645 – 5.112	0.244
Perfusor rate control	-14.283	10.665	-35.738 – 7.173	0.187
Perfusor rhythm control	0.100	9.515	-19.041 – 19.241	0.992
Balance within 24h prior bolus	-0.002	0.002	-0.005 – 0.001	0.293
Balance > 900 ml	-8.012	5.223	-18.532 – 2.508	0.132
Regular HR	11.551	4.959	1.573 – 21.528	**0.024**
Electrical cardioversion	-17.628	12.913	-43.606 – 8.350	0.179
Spontaneous conversion	9.004	5.183	-1.423 – 19.431	0.089
Pharmacological conversion	-9.827	5.066	-20.018 – 0.363	0.058
**Chest X-ray**				
Congestion prior	6.171	6.755	-7.427 – 19.768	0.366
Infiltrates prior	-4.625	5.277	-15.247 – 5.997	0.385
Effusion prior	3.125	5.969	-8.890 – 15.140	0.603
Enlargement of the cardiac silhouette prior	13.311	5.133	2.979 – 23.643	**0.013**
Congestion post	0.820	7.559	-14.434 – 16.075	0.914
Infiltrates post	4.650	5.507	-6.463 – 15.763	0.403
Effusion post	-2.197	6.377	-15.066 – 10.672	0.732
Enlargement of the cardiac silhouette post	10.232	5.527	-0.922 – 21.386	0.071
LVEF normal	0.001	0.001	0.000 – 0.002	0.086
Mean MAP 15 min prior	0.978	0.088	0.801 – 1.156	**< 0.001**

Supplementary Table S3. Differences in respiratory parameters prior- and post Landiolol bolus application in various subgroups. Data are presented as medians and interquartile ranges (IQRs) and analyzed using Wilcoxon-rank test. FiO2 = fraction of inspired oxygen, Pinsp = total inspiratory pressure, Pmean = pressure mean, PEEP = post end-expiratory pressure, RR = respiratory rate, HR = heart rate.

Suppl. Table S3	Prior Landiolol bolus	Post Landiolol bolus	p-value
**Total**			
FiO2 % (IQR)	47.5 (35-54)	50 (30-55)	0.398
Respiratory rate, /min (IQR)	25 (19-28)	25 (19-26)	0.500
Total inspiratory pressure (Pinsp), mbar (IQR)	23 (16-28)	23 (16-28)	0.258
Pressure mean (Pmean), mbar (IQR)	13 (9-15)	14 (9-16)	0.546
Post end expiratory pressure (PEEP), mbar (IQR)	8 (5-10)	8 (5-10)	0.197
**Male**			
FiO2 % (IQR)	40 (29-50)	35 (27-50)	0.786
Respiratory rate, /min (IQR)	25 (14-27)	25 (19-27)	1.000
Total inspiratory pressure (Pinsp), mbar (IQR)	21 (16-24)	20 (16-23)	0.144
Pressure mean (Pmean), mbar (IQR)	12 (9-14)	12 (9-15)	0.414
Post end expiratory pressure (PEEP), mbar (IQR)	7 (5-9)	6 (5-9)	0.317
**Female**			
FiO2 % (IQR)	50 (44-55)	50 (40-55)	0.285
Respiratory rate, /min (IQR)	26 (21-40)	24 (19-26)	0.655
Total inspiratory pressure (Pinsp), mbar (IQR)	27 (21-28)	28 (25-30)	0.785
Pressure mean (Pmean), mbar (IQR)	14 (12-16)	16 (10-16)	0.854
Post end expiratory pressure (PEEP), mbar (IQR)	10 (8-10)	10 (8-10)	0.414
**Regular HR**			
FiO2 % (IQR)	40 (25-50)	35 (25-55)	0.916
Respiratory rate, /min (IQR)	25 (20-32)	25 (22-32)	0.655
Total inspiratory pressure (Pinsp), mbar (IQR)	21 (15-26)	19 (16-24)	0.144
Pressure mean (Pmean), mbar (IQR)	12 (9-15)	12 (9-15)	1.000
Post end expiratory pressure (PEEP), mbar (IQR)	8 (7-10)	8 (5-10)	0.180
**Irregular HR**			
FiO2 % (IQR)	50 (40-55)	50 (40-55)	0.180
Respiratory rate, /min (IQR)	25 (16-27)	24 (17-25)	0.285
Total inspiratory pressure (Pinsp), mbar (IQR)	26 (20-29)	27 (19-29)	0.892
Pressure mean (Pmean), mbar (IQR)	14 (12-16)	15 (11-16)	0.414
Post end expiratory pressure (PEEP), mbar (IQR)	8 (5-10)	9 (5-10)	1.000
**Fluid balance < 900ml**			
FiO2 % (IQR)	40 (29-50)	40 (29-51)	1.000
Respiratory rate, /min (IQR)	26 (15-35)	26 (17-36)	0.317
Total inspiratory pressure (Pinsp), mbar (IQR)	21 (14-26)	21 (14-27)	0.317
Pressure mean (Pmean), mbar (IQR)	12 (9-15)	13 (9-15)	0.317
Post end expiratory pressure (PEEP), mbar (IQR)	8 (6-10)	8 (5-10)	1.000
**Fluid balance > 900ml**			
FiO2 % (IQR)	50 (38-55)	50 (34-66)	0.500
Respiratory rate, /min (IQR)	25 (19-26)	25 (22-25)	0.715
Total inspiratory pressure (Pinsp), mbar (IQR)	25 (17-28)	24 (17-28)	0.206
Pressure mean (Pmean), mbar (IQR)	13 (11-16)	14 (9-16)	0.671
Post end expiratory pressure (PEEP), mbar (IQR)	8 (5-10)	8 (5-10)	0.197

Supplementary Table S4. Differences in blood pressure prior- and post Landiolol bolus application (subdivided in 5 minutes, 15 minutes and 60 to 90 minutes before and after application). Data are presented as medians and interquartile ranges (IQRs) and analyzed using Wilcoxon-rank test. The minimum blood pressure 5, 15 and 90 minutes after the bolus application is presented in a descriptive manner. Min = minutes, Sys = Systolic blood pressure, Dia = diastolic blood pressure, MAP = mean arterial pressure, IQR = interquartile range.

Suppl. Table S4. BP response in the overall cohort

	5 min prior bolus	5 min post bolus	p-value	15 min prior bolus	15 min post bolus	p-value	60 min prior bolus	90 min post bolus	p-value
Sys, mmHg (IQR)	139 (117-160)	134 (117-153)	**0.035**	136 (119-155)	133 (112-157)	**<0.001**	135 (123-156)	132 (106-152)	**0.011**
Dia, mmHg (IQR)	73 (63-84)	69 (61-85)	0.083	72 (61-84)	69 (60-84)	**0.022**	70 (60-84)	68 (61-77)	0.427
MAP, mmHg (IQR)	93 (84-107)	93 (79-109)	**0.040**	93 (82-105)	93 (77-105)	**0.015**	90 (81-107)	89 (77-99)	0.060

Minimum BP 5 min post bolus

Sys, mmHg (IQR): 126 (109-150)
Dia, mmHg (IQR): 67 (59-81)
MAP, mmHg (IQR): 87 (75-136)

Minimum BP 15 min post bolus

Sys, mmHg (IQR): 120 (102-136)
Dia, mmHg (IQR): 64 (57-75)
MAP, mmHg (IQR): 83 (71-97)

Minimum BP 90 min post bolus

Sys, mmHg (IQR): 106 (84-120)
Dia, mmHg (IQR): 56 (48-66)
MAP, mmHg (IQR): 72 (59-84)

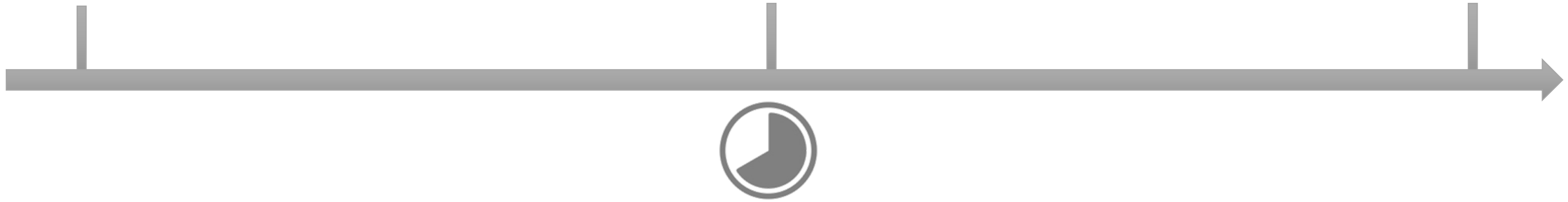

Supplementary Table S5. Differences in blood pressure prior- and post Landiolol bolus application (subdivided in 5 minutes, 15 minutes and 60 to 90 minutes before and after application) in the subgroup gender. Data are presented as medians and interquartile ranges (IQRs) and analyzed using Wilcoxon-rank test. The minimum blood pressure 5, 15 and 90 minutes after the bolus application is presented in a descriptive manner. Min = minutes, Sys = Systolic blood pressure, Dia = diastolic blood pressure, MAP = mean arterial pressure, IQR = interquartile range.

	**Suppl. Table S5. BP response in gender**									
	gender	5 min prior bolus	5 min post bolus	p-value	15 min prior bolus	15 min post bolus	p-value	60 min prior bolus	90 min post bolus	p-value
Sys,mmHg (IQR)	male	148 (117-170)	135 (112-156)	**0.017**	148 (119-167)	136 (113-159)	**< 0.001**	140 (122-163)	133 (111-163)	**0.018**
	female	132 (118-151)	130 (117-154)	0.465	132 (120-149)	130 (111-153)	0.140	132 (124-145)	123 (103-151)	0.218
Dia, mmHg (IQR)	male	82 (69-90)	79 (62-87)	**0.023**	82 (69-87)	78 (66-85)	**0.003**	76 (63-89)	76 (64-83)	0.151
	female	64 (62-73)	66 (60-75)	0.948	65 (60-72)	65 (58-77)	0.770	61 (58-72)	63 (59-68)	0.613
MAP,mmHg (IQR)	male	104 (84-116)	98 (74-110)	**0.008**	104 (83-111)	100 (77-112)	**0.002**	99 (82-113)	93 (78-110)	**0.037**
	female	88 (81-98)	87 (79-106)	0.843	88 (82-98)	87 (77-101)	0.738	89 (80-93	85 (76-98)	0.697

	**Minimum BP 5 min post bolus**
Sys, mmHg (IQR)	
male	132 (107-154)
female	122 (111-140)
Dia, mmHg (IQR	
male	77 (68-84)
female	62 (57-74)
MAP, mmHg (IQR)	
male	96 (73-108)
female	83 (75-94)

	**Minimum BP 15 min post bolus**
Sys, mmHg (IQR)	
male	123 (105-140)
female	116 (97-132)
Dia, mmHg (IQR	
male	69 (60-80)
female	60 (52-72)
MAP, mmHg (IQR)	
male	87 (71-98)
female	80 (70-89)

	**Minimum BP 90 min post bolus**
Sys, mmHg (IQR)	
male	107 (81-120)
female	104 (84-120)
Dia, mmHg (IQR	
male	63 (50-68)
female	53 (47-59)
MAP, mmHg (IQR)	
male	78 (58-85)
female	69 (61-80)

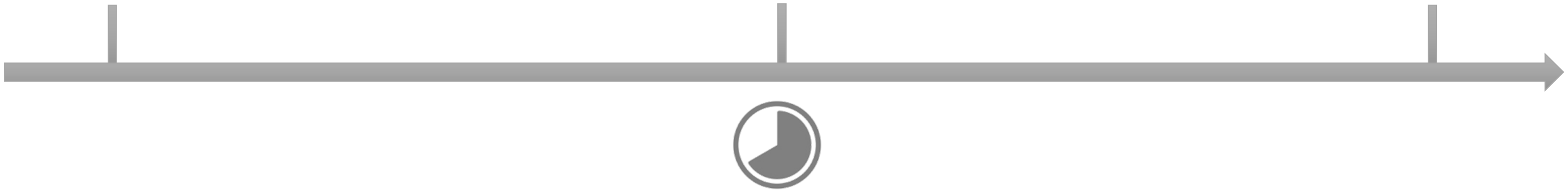

Supplementary Table S6. Differences in blood pressure prior- and post Landiolol bolus application (subdivided in 5 minutes, 15 minutes and 60 to 90 minutes before and after application) in the subgroup of regular and irregular tachycardia before the bolus . Data are presented as medians and interquartile ranges (IQRs) and analyzed using Wilcoxon-rank test. The minimum blood pressure 5, 15 and 90 minutes after the bolus application is presented in a descriptive manner. RT = regular tachycardia, IRT = irregular tachycardia, Min = minutes, Sys = Systolic blood pressure, Dia = diastolic blood pressure, MAP = mean arterial pressure, IQR = interquartile range.

	**Suppl. Table S6. BP response in regular and irregular tachycardia**									
	tachycardia	5 min prior bolus	5 min post bolus	p-value	15 min prior bolus	15 min post bolus	p-value	60 min prior bolus	90 min post bolus	p-value
Sys,mmHg (IQR)	RT	148 (124-170)	146 (124-162)	0.313	148 (127-159)	148 (124-159)	0.057	144 (124-163)	137 (115-154)	**0.030**
	IRT	132 (113-155)	122 (98-144)	**0.037**	127 (117-151)	117 (103-149)	**0.004**	128 (118-145)	130 (99-151)	0.118
Dia, mmHg (IQR)	RT	81 (65-91)	84 (71-88)	0.614	82 (69-87)	78 (66-85)	**0.003**	82 (68-89)	76 (62-84)	0.180
	IRT	68 (63-79)	66 (60-69)	0.064	65 (60-72)	65 (58-77)	0.770	63 (58-72)	63 (59-73)	0.713
MAP,mmHg (IQR)	RT	104 (86-116)	102 (87-112)	0.180	104 (83-110)	102 (86-111)	0.131	102 (87-114)	95 (80-104)	0.057
	IRT	88 (81-101)	84 (74-84)	0.110	88 (82-94)	84 (72-98)	0.050	86 (79-93)	86 (74-94)	0.517

	**Minimum BP 5 min post bolus**
Sys, mmHg (IQR)	
RT	136 (117-153)
IRT	120 (96-138)
Dia, mmHg (IQR	
RT	79 (61-85)
IRT	63 (57-69)
MAP, mmHg (IQR)	
RT	98 (81-108)
IRT	82 (72-94)

	**Minimum BP 15 min post bolus**
Sys, mmHg (IQR)	
RT	126 (115-141)
IRT	110 (92-132)
Dia, mmHg (IQR	
RT	72 (60-79)
IRT	61 (52-67)
MAP, mmHg (IQR)	
RT	87 (71-98)
IRT	80 (70-89)

	**Minimum BP 90 min post bolus**
Sys, mmHg (IQR)	
RT	107 (93-120)
IRT	96 (74-122)
Dia, mmHg (IQR	
RT	63 (52-68)
IRT	52 (47-64)
MAP, mmHg (IQR)	
RT	78 (66-84)
IRT	67 (55-80)

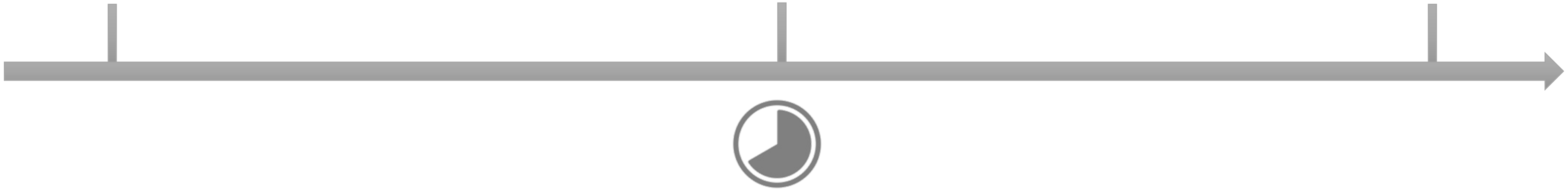

Supplementary Table S7. Differences in blood pressure prior- and post Landiolol bolus application (subdivided in 5 minutes, 15 minutes and 60 to 90 minutes before and after application) in the subgroups fluid balance above 900 ml und below 900 ml. Data are presented as medians and interquartile ranges (IQRs) and analyzed using Wilcoxon-rank test. The minimum blood pressure 5, 15 and 90 minutes after the bolus application is presented in a descriptive manner. Min = minutes, Sys = Systolic blood pressure, Dia = diastolic blood pressure, MAP = mean arterial pressure, IQR = interquartile range.

	**Suppl. Table S7. BP response in fluid balance**									
	Fluid Balance	5 min prior bolus	5 min post bolus	p-value	15 min prior bolus	15 min post bolus	p-value	60 min prior bolus	90 min post bolus	p-value
Sys,mmHg (IQR)	> 900ml	143 (115-160)	128 (115-153)	**0.002**	139 (118-159)	130 (110-158)	**< 0.001**	132 (122-157)	132 (102-153)	0.053
	< 900ml	137 (126-155)	141 (118-162)	0.711	136 (121-154)	138 (117-159)	0.324	136 (124-160)	136 (116-151)	0.227
Dia, mmHg (IQR)	> 900ml	79 (63-83)	68 (61-80)	**0.011**	69 (61-82)	68 (59-77)	0.054	65 (59-80)	66 (59-77)	0.942
	< 900ml	77 (66-85)	83 (65-87)	0.727	79 (69-87)	79 (63-86)	0.285	74 (61-86)	76 (63-83)	0.727
MAP,mmHg (IQR)	> 900ml	88 (82-105)	85 (79-106)	**0.003**	87 (81-105)	85 (75-103)	**0.043**	87 (81-105)	87 (76-98)	0.227
	< 900ml	98 (88-110)	103 (88-111)	0.679	98 (88-111)	101 (87-111)	0.398	94 (87-108)	94 (80-102)	0.316

	**Minimum BP 5 min post bolus**
Sys, mmHg (IQR)	
> 900ml	121 (106-150)
< 900ml	132 (116-151)
Dia, mmHg (IQR	
> 900ml	65 (58-78)
< 900ml	78 (60-84)
MAP, mmHg (IQR)	
> 900ml	83 (74-101)
< 900ml	96 (76-108)

	**Minimum BP 15 min post bolus**
Sys, mmHg (IQR)	
> 900ml	111 (96-136)
< 900ml	126 (116-140)
Dia, mmHg (IQR	
> 900ml	63 (55-73)
< 900ml	70 (59-80)
MAP, mmHg (IQR)	
> 900ml	79 (69-94)
< 900ml	87 (76-99)

	**Minimum BP 90 min post bolus**
Sys, mmHg (IQR)	
> 900ml	103 (79-117)
< 900ml	108 (92-123)
Dia, mmHg (IQR	
> 900ml	53 (47-64)
< 900ml	59 (52-68)
MAP, mmHg (IQR)	
> 900ml	68 (55-80)
< 900ml	78 (66-85)

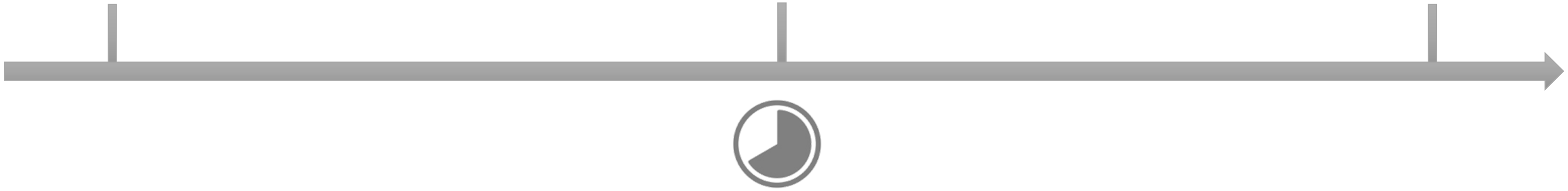

Supplementary Table S8. Differences in diagnostic imaging (X-ray) prior- and post-Landiolol bolus and latest cardiac function (LVEF) prior Landiolol bolus in various subgroups. The fluid balance accounts for the overall balance right before push-dose Landiolol application. “RT” and “IRT” concerns the heart rhythm before bolus application. Data are presented as medians and interquartile ranges (IQRs) and analyzed using Kruskal–Wallis test. RT = regular tachycardia, IRT = irregular tachycardia, LVEF = Left ventricular ejection fraction, ASAT = aspartate-aminotransferase, ALAT = alanine-aminotransferase, MCV = mean corpuscular volume, MCH = mean corpuscular hemoglobin, MCHC = mean cell hemoglobin concentration, Hct = haematocrit, CRP = C-reactive protein.

Suppl. Table S8	Total	Male	Female	RT	IRT	Fluid balance <900 ml	Fluid balance >900 ml
Diagnostic imaging							
Chest x-ray prior bolus, n (%)	48 (98)	25 (96.2)	23 (100)	21 (95.5)	27 (100)	19 (100)	28 (100)
Congestion, n (%)	9 (18.4)	2 (7.7)	7 (30.4)	3 (13.6)	6 (22.2)	5 (26.3)	4 (14.3)
infiltrates, n (%)	24 (49)	9 (34.6)	15 (65.2)	9 (40.9)	15 (55.6)	12 (63.2)	12 (42.9)
effusion, n (%)	13 (26.5)	7 (26.9)	6 (26.1)	3 (13.6)	10 (37.0)	4 (21.1)	9 (32.1)
Enlargement of the cardiac silhouette, n (%)	18 (36.7)	8 (30.8)	10 (43.5)	9 (40.9)	9 (33.3)	11 (57.9)	7 (25.0)
Chest x-ray post bolus	44 (89.8)	22 (84.6)	22 (95.7)	19 (86.4)	25 (92.6)	17 (89.5)	26 (92.9)
Congestion, n (%)	7 (14.3)	1 (3.8)	6 (26.1)	2 (9.1)	5 (18.5)	4 (21.1)	3 (10.7)
infiltrates, n (%)	20 (40.8)	10 (38.5)	10 (43.5)	9 (40.9)	11 (40.7)	12 (63.2)	8 (28.6)
effusion, n (%)	11 (22.4)	4 (15.4)	7 (30.4)	3 (13.6)	8 (29.6)	4 (21.1)	7 (25.0)
Enlargement of the cardiac silhouette, n (%)	16 (32.7)	6 (23.1)	10 (43.5)	6 (27.3)	10 (37.0)	10 (52.6)	6 (21.4)
LVEF previous							
Normal, n (%)	16 (32.7)	7 (26.9)	9 (39.1)	4 (18.2)	12 (44.4)	2 (10.5)	13 (46.4)
Mild dysfunction, n (%)	0 (0)	0 (0)	0 (0)	0 (0)		0 (0)	0 (0)
Moderate dysfunction, n (%)	2 (4.1)	1 (3.8)	1 (4.3)	1 (4.5)	1 (3.7)	1 (5.3)	1 (3.6)
Severe dysfunction, n (%)	4 (8.2)	2 (7.7)	2 (8.7)	1 (4.5)	3 (11.1)	1 (5.3)	3 (10.7)
Serum laboratory results prior							
Platelet, G/L	238 (157-351)	243 (123-303)	238 (159-458)	275,5 (141,75-374)	238 (157-356)	275 (157-346)	238 (157,5-374,75)
Leucocyte, G/L	11.56 (8.13-17.88)	11.3 (6.4-14.4)	11.56 (9.97-20.95)	11,27 (6,37-13,25)	11,56 (9-20,95)	9,38 (6,42-12,78)	12,39 (9,59-20,95)
Erythrocytes, G/L	3.21 (2.98-3.54)	3.2 (3.0-3.5)	3.25 (2.97-3.62)	3,21 (3,01-3,46)	3,33 (2,97-3,61)	3,2 (2,86-3,36)	3,21 (3,13-3,59)
Hemoglobin, g/dL	9.7 (8.9-10.5)	9.7 (9.3-10.7)	9.8 (8.1-10.4)	9,4 (9,15-10,2)	10 (8,6-10,9)	9,3 (8,7-9,7)	10 (8,75-10,35)
Hct, %	29.4 (26.8-31.8)	29.2 (27.2-33.0)	29.6 (24.7-31-1)	28,7 (27,15-30,25)	29,8 (26,5-33,5)	28,4 (26,5-29,4)	29,6 (26,18-31,1)
MCV, fL	90.5 (85.9-94.4)	92.7 (86.5-94.7)	90.4 (81-92.8)	90,6 (86,08-93,83)	90,4 (85,3-96,6)	92,1 (86,6-96,5)	90,4 (84,65-93,78)
MCH, pg	29.4 (28.3-30.7)	30.4 (19.0-32-3)	29.1 (26.2-29.9)	29,25 (28,03-30,55)	29,6 (29,1-31,9)	29,7 (28,3-32,9)	29,3 (28,4-30,65)
MCHC, g/dL	32.7 (32.2-33.9)	33.2 (32.5-34.2)	32.5 (32.2-33.3)	32,6 (32,18-34,13)	33 (32,2-33,7)	32,5 (31,3-34,2)	33,05 (32,2-33,9)
Fibrinogen, g/L	4.6 (3.6-5.9)	4.7 (4.0-7.0)	4.6 (2.5-5.7)	5,37 (4,23-6,11)	4,23 (2,93-5,67)	4,23 (3,56-6,11)	5,02 (3,1-6,53)
CRP, mg/l	74.7 (34.6-111.0)	66.1 (25-104.8)	84.3 (52.3-139)	81 (24,75-121,75)	70,5 (48,9-92,6)	52,3 (25-106)	84,3 (60,7-144,5)
Procalcitonin, ng/ml	0.37 (0.16-0.52)	0.21 (0.16-0.54)	0.37 (0.32-0.46)	0,21 (0,16-0,37)	0,42 (0,17-0,71)	0,18 (0,15-0,39)	0,37 (0,22-0,54)
Sodium, mmol/L	142 (137-144)	143 (139-143)	141 (136-145)	142 (137-143)	142 (137-145)	142 (139-143)	142 (137-145)
Potassium, mmol/L	4.1 (3.8-4.2)	4 (3.7-4.2)	4.1 (3.8-4.4)	4 (3,8-4,2)	4,1 (3,65-4,63)	3,9 (3,8-4,4)	4,1 (3,7-4,2)

Chloride, mmol/L	105 (100-109)	105 (100-109)	105 (97-107)	104,5 (100-110)	105 (98-108)	104 (100-109)	105,5 (99,25-110)
Calcium, mmol/L	2.05 (1.94-2.09)	2.05 (1.95-2.09)	2.05 (1.93-2.09)	2,05 (1,93-2,09)	2,05 (1,95-2,11)	2,05 (1,95-2,08)	2,05 (1,93-2,12)
Magnesium, mmol/L	0.9 (0.75-1.00)	0.80 (0.60-0.93)	0.9 (0.8-1.0)	0,8 (0,6-0,9)	0,9 (0,8-1,1)	0,8 (0,6-0,9)	0,9 (0,8-1,08)
Creatinine, mg/dL	0.8 (0.6-1.0)	0.8 (0.6-1.0)	0.8 (0.5-1.9)	0,7 (0,6-0,9)	1 (0,7-1,5)	0,6 (0,5-1)	0,8 (0,7-1)
ASAT, U/L	34.5 (23.5-59.8)	43 (27-59)	33 (21-193)	34,5 (21-50,5)	38,5 (25,25-133,75)	29 (20-52)	45 (33-114)
ALAT, U/L	48 (22-82)	57 (29-78)	35 (20-217)	47 (31-72,5)	54 (20-176)	40 (22-58)	54,5 (23,5-158,5)
Triglycerides, mg/dL	147 (121-240)	144 (100-231)	166 (132-289)	186,5 (124-299,5)	144 (101-210)	153 (124-289)	144 (110,5-219,5)
Cholesterol, mg/dL	154 (120-196)	169 (118-195)	153 (120-199)	180 (148-212,5)	128 (113-156)	182 (130-206)	148 (120-165,75)
Serum laboratory results post							
Platelet, G/L	260 (152-373.5)	258 (143-332)	338 (198-435)	258 (138-409)	279 (166-345)	247 (166-383)	280 (138-424)
Leucocyte, G/L	10.65 (7.76-16.84)	9.9 (6.7-15.5)	13.22 (9.11-18.49)	9,87 (6,74-13,24)	15,17 (7,92-17,84)	8,82 (6,81-13,22)	15,17 (9,15-18,49)
Erythrocytes, G/L	3.25 (2.94-3.67)	3.3 (3.1-3.6)	3.25 (2.84-3.72)	3,21 (2,97-3,4)	3,42 (2,9-3,72)	3,12 (2,81-3,63)	3,26 (3,21-3,68)
Hemoglobin, g/dL	9.6 (8.7-10.6)	9.7 (9.2-10.4)	8.8 (8.3-10.6)	9,3 (8,7-9,9)	9,9 (8,6-10,6)	9,2 (8,6-10,4)	9,7 (8,6-10,6)
Hct, %	29.1 (27.1-32.8)	29.7 (27.2-31.1)	28 (24.8-33.3)	28,5 (26,85-30,05)	30,8 (27,2-33,3)	27,8 (27,2-30,8)	29,7 (25,6-33,3)
MCV, fL	90.2 (86.2-95.3)	93.0 (86.3-95.5)	89.5 (81.9-95.1)	91,8 (85,55-94,45)	89,5 (86,2-95,6)	93 (86,3-96,8)	89,5 (84,3-95,1)
MCH, pg	29.3 (28.5-30.6)	30.1 (28.9-31.6)	28.6 (26.2-29.7)	29,2 (27,95-30,45)	29,3 (28,5-31,5)	29,5 (28,7-30,6)	29,2 (28,5-30,5)
MCHC, g/dL	32.8 (32.2-33.7)	33.1 (32.6-33.8)	32.5 (31.9-33.2)	32,6 (32,05-33,8)	32,9 (32,2-33,6)	32,3 (31,9-33,8)	32,9 (32,6-33,6)
Fibrinogen, g/L	4.7 (3.8-5.5)	4.8 (3.9-5.9)	4.56 (3.18-5.48)	4,76 (4,04-5,29)	4,58 (3,33-5,98)	4,71 (3,56-5,52)	4,76 (3,78-5,98)
CRP, mg/l	76.5 (49.3-117.0)	54.4 (30.8-97.1)	95 (60.3-117)	78,1 (40,25-97,05)	74,8 (50,5-138)	48,9 (28,6-96,7)	92,3 (54,4-160)
Procalcitonin, ng/ml	0.44 (0.19-0.72)	0.3 (0.16-0.59)	0.45 (0.37-5.49)	0,27 (0,14-0,45)	0,58 (0,38-5,49)	0,27 (0,16-0,56)	0,51 (0,38-2,12)
Sodium, mmol/L	141 (138-143)	142 (140-143)	140 (136-141)	142 (137-143)	141 (139-143)	140 (137-143)	141 (140-142)
Potassium, mmol/L	4 (3.7-4.7)	3.9 (3.7-4.3)	4.2 (3.8-5.3)	3,85 (3,7-4,18)	4,25 (3,75-5,3)	3,9 (3,6-4,1)	4,2 (3,75-5)
Chloride, mmol/L	105 (100.3-108)	105 (101.5-108)	103 (95-109)	103 (101-110)	105 (97-108)	103 (101-106)	107 (97-109)
Calcium, mmol/L	2.05 (1.94-2.13)	2.08 (1.96-2.13)	2.01 (1.93-2.11)	2,01 (1,95-2,11)	2,11 (1,93-2,19)	2,08 (1,95-2,13)	2,01 (1,93-2,12)
Magnesium, mmol/L	0.9 (0.8-1,2)	0.80 (0.70-1.00)	1.1 (0.8-1.2)	0,8 (0,7-0,9)	1,1 (0,9-1,2)	0,8 (0,7-1)	1 (0,8-1,2)
Creatinine, mg/dL	0,85 (0.53-1.40)	0.80 (0.55-0.95)	1.0 (0.5-1.6)	0,6 (0,5-0,9)	1,1 (0,7-1,6)	0,7 (0,5-1,3)	0,9 (0,6-1,4)
ASAT, U/L	40 (23-95)	41 (27-59)	36 (21-141)	37 (17,75-54)	42 (33,5-123)	27 (16-39)	59 (36-109)
ALAT, U/L	53.5 (19.0-97.0)	60 (34-93.5)	32 (17-230)	49 (29-87)	59 (17-159)	37 (26-65)	84 (17-115)
Triglycerides, mg/dL	154 (118-243)	141 (109-226)	154 (131-253)	206 (137,5-286,5)	143 (107,25-187)	175,5 (137,25-255,75)	143 (109-209)
Cholesterol, mg/dL	148 (123-197)	181 (127.5-214.0)	147 (122-193)	189 (141,5-214,5)	137 (121,25-185)	195 (133-214,25)	145 (122-181)
